# Detection of clade 2.3.4.4b H5N1 high pathogenicity avian influenza virus in a sheep in Great Britain, 2025

**DOI:** 10.1080/22221751.2025.2547730

**Published:** 2025-09-15

**Authors:** Ashley C. Banyard, Holly Coombes, Jacob Terrey, Natalie McGinn, James Seekings, Benjamin Clifton, Benjamin C. Mollett, Cecilia Di Genova, Pia Sainz-Dominguez, Laura Worsley, Raquel Jorquera, Elizabeth Billington, Edward Fullick, Audra-Lynne Schlachter, David Jorge, Alejandro Núñez, Marco Falchieri, Joe James, Scott M. Reid

**Affiliations:** a Influenza and Avian Virology Team, Department of Virology, Animal and Plant Health Agency (APHA-Weybridge), Addlestone, UK; b WOAH/FAO International Reference Laboratory for Avian Influenza, Animal and Plant Health Agency (APHA-Weybridge), Addlestone, UK; c The National Emergency Epidemiology Group (NEEG), Animal and Plant Health Agency (APHA-Weybridge), Addlestone, UK; d APHA Field Epidemiology Team, APHA Bridgwater, Bridgwater, UK; e APHA Thirsk Veterinary Investigation Centre, Thirsk, UK; f Department of Pathology and Animal Sciences, Animal and Plant Health Agency (APHA-Weybridge), Addlestone, UK

**Keywords:** High pathogenicity, avian influenza, sheep, lactating mammals, ruminant infection, emergence

## Abstract

Clade 2.3.4.4b H5N1 high pathogenicity avian influenza virus (HPAIV) continues to pose a significant global threat, affecting wild and domestic avian, and mammalian species. In early 2024, H5N1 HPAIV was detected in dairy cattle in the United States of America, where it has continued to circulate, with sporadic detections also reported in other ruminant species. The detection of high viral loads in milk from infected cattle, resulted in several human infections, underscoring the zoonotic potential of these viruses. In response, several countries have intensified surveillance in non-avian species to evaluate the potential for undetected viral circulation in captive mammals. In Great Britain, bulk milk tank testing of cattle and targeted surveillance of captive mammalian species on an infected premises is undertaken in accordance with the outcome of a rapid risk assessment. This assessment is undertaken to determine epidemiological links between the poultry and captive mammals. A result of this testing was the first recorded detection of clade 2.3.4.4b H5N1 HPAIV in a sheep in March 2025, identified on an infected poultry premises housing ducks, chickens, turkeys and geese in Great Britain. An initial seropositive result in a single ewe triggered further investigation, confirming serological positivity across repeated sampling and the presence of viral RNA in milk samples. This detection was confined to a single animal and is likely attributable to proximity to infected poultry and a presumed heavily contaminated environment. The implications of this detection in a ruminant host are discussed in the context of interspecies transmission and surveillance strategies.

## Introduction

Clade 2.3.4.4b H5N1 high pathogenicity avian influenza virus (HPAIV) continues to cause significant infection and mortalities in both wild birds and poultry globally [1,2]. The impact of the H5N1 panzootic has been profound, with outbreaks decimating wild bird populations, the poultry sector, and populations of terrestrial and marine mammals where spillover has occurred [2,3]. High mortality rates are frequently observed among wild animals, significantly affecting avian species including those of conservation concern [2,4]. Wild bird mortalities increase environmental pressure which in turn increases the risk of infection for wild scavenging species, including terrestrial and aquatic mammals [3]. Alongside the infection of wildlife, infection of captive mammals has also been reported in zoological collections, household settings and farming sectors. Such outbreaks have included captive bush dogs [5], farmed mink and foxes in Europe [6,7], household cats [8], and most notably outbreaks in farmed dairy cattle in the United States of America (USA) [9,10].

From a ruminant perspective, H5N1 HPAIV (genotype B3.13) was first reported in dairy cattle in the USA on 25th March 2024 [11]; however, phylogenetic analysis indicates initial spillover from wild birds into cattle in late 2023. Since this detection, B3.13 has continued to spread in cattle, forming a discrete lineage, yet apparently becoming extinct in wild birds [12]. The widespread infection of dairy cattle has significantly elevated the zoonotic risk posed by H5N1 HPAIV, largely due to; (**i**) the observation of high viral titres present in milk from infected cattle, and (**ii**) concerns around the virus acquiring mammalian adaptations which may bestow increased fitness in humans [10,13,14]. While genotype B3.13 was still circulating in cattle, a second H5N1 HPAIV genotype was also reported in cattle in the state of Nevada, USA on the 31th January 2025 [15], and separately in the state of Arizona, USA on 13th February 2025 [16]. Both of these detections were with genotype D1.1 predominantly circulating in wild birds across the USA [17]. While the spillover dynamics of D1.1 remains to be elucidated, these detections clearly indicate multiple separate incursions from birds to cattle have occurred. To date, HPAIV (mainly of genotype B3.13) has been detected in 17 states in over 1000 dairy herds across the USA. Zoonotic risk has increased with detections in dairy cattle, attributable to very high titres of virus in milk of infected cattle and it is of note that of the 70 cases of human infection reported in the USA with H5N1, 41 of these have been associated with exposure to infectious material in a dairy setting [11]. Alongside dairy cattle, H5N1 has also been sporadically reported in other farmed mammals including alpacas, pigs and goats [12,18], as well as captive mammals present on farms such as cats [19]. Currently there have been no previous reported detections of H5 HPAIV in sheep globally.

To date, only two H5N1 HPAIV genotypes (B3.13 and D.1.1) have been detected in dairy cattle globally, and both are exclusively found in wild birds within the Americas (mainly the USA) [18,20]. However, experimental studies have shown that the infection of cattle is not constrained by the genetic composition of these viruses; both American and European strains can induce similar infection and disease characteristics in cattle and other mammalian species [21]. These findings, alongside the scale of H5N1 HPAIV infections in USA cattle, have driven many countries to enhance surveillance for HPAIV in mammals, particularly where there is a higher risk of exposure to infectious material on infected poultry premises. However, to date, H5N1 HPAIV has not been detected in cattle outside of the USA [22-25]. In Great Britain (GB), national bulk milk tank sampling of a representative proportion of dairy cattle herds was undertaken. Over 500 bulk-milk samples, from 455 farms across GB, were all found negative for HPAIV [26]. In addition, targeted surveillance is being undertaken for captive mammals co-located with infected poultry where the risk of exposure to HPAIV is assessed as greater than the background risk posed by wild bird populations. Thus far in GB, the detection of influenza of avian origin in mammalian species has been limited to sporadic infections of wild mammals [27], including detections in foxes and otters alongside infection of marine mammals, predominantly seal species [27]. So far there has only been a single detection in captive mammals in GB, with H5N1 HPAIV being detected in a single group of captive bush dogs within a zoological collection in November 2022 [5,28]. However, as the clade 2.3.4.4b H5N1 panzootic continues to impact both wild and captive avian species, the risk to mammals co-located with infected poultry remains.

Here, the detection of H5N1 in a backyard flock including chickens, ducks, turkeys and geese is described whereby virus detection in avian species led to sampling of mammals with the resultant detection of infection with clade 2.3.4.4b H5N1 HPAIV in a single sheep. Factors leading to this detection are described.

## Materials and methods

### Clinical investigation, post-mortem examination, tissue sampling and histopathological analysis

Samples from birds were taken as described previously [29]. Samples from a proportion (38%; n = 10/26) of the sheep were initially taken to evaluate infection status with oral and rectal swabs being taken alongside blood sampling. Later sampling included individual animal composite milk samples where serological data had indicated infection (Supplementary Table 4). Following molecular and serological detection, a full postmortem (PM) examination and extensive tissue sampling of all major organ systems was undertaken on the affected ewe. Tissue samples were split for homogenization and histopathological assessment. For the former ∼0.5 grams of each tissue was processed into Precellys Hard tissue Homogenizing Tubes (CK28; Bertin Technologies, France) and 0.5 ml of phosphate-buffered saline (PBS) was added. Samples were homogenized for 2 min at 7000 RPM using Precellys24 homogenizer (Bertin Technologies, France). Supernatants were centrifuged at 10,000 xg for 30 s, and clarified supernatants were processed for extraction of viral RNA (vRNA) for molecular analyses [30].

For histopathology, samples were fixed in 10% (v/v) neutral buffered formalin for a minimum period of 5 days before being routinely processed and embedded in paraffin wax. Twenty-two different wax blocks were prepared from mammary gland (11 for each left and right mammary gland) Four-micron thick serial sections were either stained with haematoxylin and eosin (H&E), or for immunohistochemistry (IHC), using a mouse monoclonal anti-influenza A nucleoprotein (NP) antibody (Statens Serum Institute, Copenhagen, Denmark) as described previously [30]. The overall distribution of virus-specific staining in each tissue was assessed using a previously established criteria modified from [31]. Specificity of immunolabelling was assessed using positive control sections and by replacing the primary antibody with a matching mouse IgG isotype in test sections; no non-specific cross-linking was observed. Additionally, Gram Twort staining was performed in mammary gland sections.

### Virological investigation

#### RNA extraction and molecular analysis

RNA was extracted from samples using the MagMAX CORE Nucleic Acid Purification Kit (ThermoFisher Scientific) as previously described [30] with tissues being homogenised in L-15 buffer prior to extraction. Extracted RNA from swabs and tissues was assessed for vRNA using Real-Time Reverse Transcription–Polymerase Chain Reaction (RT–PCR). RT–PCR assays used were matrix (M) gene specific [32], a H5 HPAIV specific [33] and/or NA specific [34,35] gene. RT–PCR Cq values < 36.00 were considered as AIV positive. Samples with Cq ≥36 were considered negative [30].

#### Attempted virus isolation

For virus isolation, 100 µl of the sample material was diluted with 100 µl of PBS and inoculated into the allantoic cavity of three specific pathogen-free (SPF) embryonated chicken eggs (ECEs), following established protocols [36,37]. At two days post-inoculation (dpi), the allantoic fluid from one ECE was tested for the presence of virus using the haemagglutination assay as previously described [36]. Fluids exhibiting haemagglutination activity ≥16 were considered positive, whereas those with activity <16 were classified as negative. Allantoic fluids testing negative at 2 dpi were subjected to a second passage (passage 2) by inoculation into additional ECEs under the same conditions. At six dpi, allantoic fluid from all ECEs was harvested and tested by HA. Samples yielding negative haemagglutination activity following this protocol were deemed negative for infectious virus [45].

Attempts were also made to concentrate virus present in milk by the addition of chicken red blood cells (cRBCs), following a previously published protocol [38]. Briefly, 10 µl of packed cRBCs were added to 1 ml of milk collected from the positive sheep and mixed by gentle inversion. The samples were incubated at 4 °C for 1 hour, after which the cells were pelleted by centrifugation at 1500 rpm for 5 minutes at 4 °C. The resulting cell pellet was resuspended in 200 µl of Leibovitz medium (LM) and used for virus isolation by inoculation into ECEs as described above.

#### Serological assessment

Serum was separated from blood samples and heated at 56 °C for 30 min before being treated with receptor destroying enzyme (RDE) as described previously [39]. Hemagglutination inhibition assays were performed on processed serum samples as described previously using A/Teal/England/7394-2805/2005 (H5N3), A/chicken/Scotland/1959 (H5N1), A/Chicken/Wales/053969/2021 (H5N1) (clade 2.3.4.4b H5N1) antigens all at 4 hemagglutination units [36]. In addition, batches of serum which included an HI positive sample were run on two commercial ELISA tests were used as per manufacturer’s instructions. These included (**i**) ID Screen® Influenza A Antibody Competition Multi-species ELISA (ID Screen® Influenza A Antibody Competition Multi-species – Innovative Diagnostics), a competitive ELISA for the detection of antibodies against the NP of the Influenza A virus in multiple species and (**ii**) the ID Screen® Influenza H5 Antibody Competition 3.0 Multi-species ELISA (ID Screen® Influenza H5 Antibody Competition 3.0 Multi-species – Innovative Diagnostics), a multi-species competitive ELISA for the detection of antibodies against the H5 hemagglutinin of the Influenza A virus. All ELISAs were undertaken as per manufacturer’s instructions. Sheep sera were diluted 1/5 through addition to receptor destroying enzyme (RDE).

#### Genomic analysis

Viral genome sequences were generated from the composite milk sample using Oxford Nanopore Technology, by adapting a method previously described [40,41]. For all avian samples extracted vRNA was converted to double stranded cDNA and amplified using a one-step RT–PCR using SuperScript III One-Step RT–PCR kit (Thermo Fisher Scientific). Extracted RNA from the composite milk sample was converted to double stranded cDNA and amplified in triplicate. All three aliquots of amplified cDNA were pooled and concentrated using an ethanol precipitation. Briefly, 5 µL 5M NaCl and 1 µL glycogen was added to 50 µL cDNA and mixed by vortexing. Then 165 µL of 100% ethanol was added and the sample incubated at −20°C for a minimum of 30 minutes. The cDNA was pelleted by centrifugation at 1500 rpm for 5 minutes at 4 °C. The resulting pellet was washed with 70% ethanol and resuspended in sterile H_2_O.

Due to difficulty in generating complete sequences for the milk polymerase acidic (PA), polymerase basic 1 (PB1) and haemagglutinin (HA) segments from the initial sequencing attempts, these segments were converted to cDNA and amplified individually using segment specific primers (Supplementary Table 1).

Amplified cDNA was purified with Agencourt AMPure XP beads (Beckman Coultrer) prior to sequencing library preparation using the Native Barcoding Kit (Oxford Nanopore Technologies) and sequenced using a GridION Mk1 (Oxford Nanopore Technologies), according to manufacturer’s instructions.

Assembly of the influenza A viral genomes was performed using a custom in-house pipeline publicly available here (APHA-VGBR/WGS_Pipelines/denovoAssembly_ONT_Public.sh). Raw reads from the composite milk, PB1, PA and HA samples were combined prior to assembly. Comparison of the study-derived sequences and contemporary H5 sequences was undertaken against all avian H5 sequences available on GISAID between 1st January 2020 and 23rd April 2025. All sequences were aligned on a per segment basis using Mafft v7.520 [42] and trimmed against a reference using SeqKit v2.5.1 [4]. The trimmed alignments were used to a infer maximum-likelihood phylogenetic tree IQ-Tree version 2.2.3 [43] along with ModelFinder [40] and 1,000 ultrafast bootstraps [42]. Ancestral sequence reconstruction and inference of molecular-clock phylogenies were performed using TreeTime v0.10.1 [44]. Phylogenetic trees were visualized using R version 4.3.3 with ggplot2 [45] and ggtree version 3.14.0 [46]. Sequences were genotyped from phylogenetic trees by comparison to known reference sequences for all genotypes currently circulating in the UK.

Sequences derived from both sheep and associated poultry outbreak were assessed for the presence of adaptive mutations that may confer increased replication in mammals. All sequences were aligned on a per segment basis using MAFFT v7.520 [42] and manually trimmed to the open reading frame using Aliview version 1.28 [47]. Trimmed sequences were translated to amino acids and visually inspected for mutations. All influenza sequences generated in this study are available through the GISAID EpiFlu Database (https://www.gisaid.org, Supplementary Table 4).

## Results

**Figure 1.**
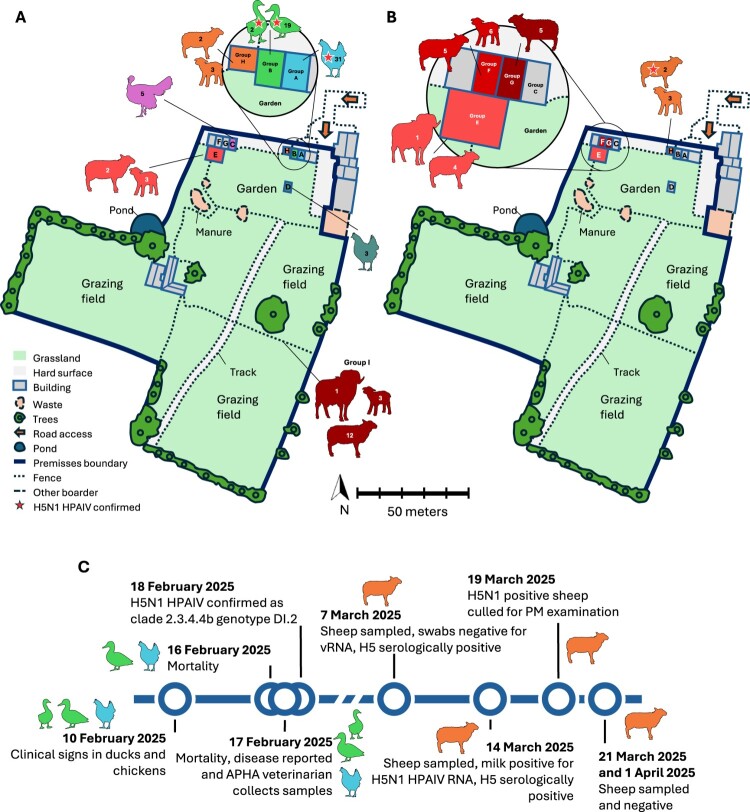
Map of the infected poultry premises with H5N1 HPAIV positive sheep and timeline of key events. Map of the infected poultry premises indicating major geographical features and animal location and numbers during veterinary visits by APHA (A) at the time of poultry sampling (17th February 2025), (B) at the time of initial sheep sampling (7th March 2025). (C) A timeline of key events during the infection event.

On 18^th^ February 2025 HPAI H5N1 was confirmed on a backyard mixed poultry holding in Yorkshire. The infected premises was a flock (n = 60 poultry) consisting of 34 chickens (*Gallus gallus domesticus*), 5 turkeys (*Meleagris gallopavo*), 19 ducks (*Anas platyrhynchos domesticus*) and 2 geese (*Anser anser domesticus*) (Figure 1a and b). The birds had been housed since the housing order came into effect on 23rd December 2024 as the infected premises lies within the Avian Influenza Prevention Zone (AIPZ) [48]. The poultry were housed in four groups; Group A comprised 31 chickens in a stable, Group B comprised 19 ducks and two geese in another stable, Group C comprised five turkeys in another shed, and Group D comprised 3 chickens in a wooden coop in the garden. Additionally, the premises had one household cat (*Felis catus*) and 26 sheep (*Ovis aries*) (16 breeding ewes, 9 lambs and 1 ram). At the time of the report case (Figure 1a), one group of sheep were housed next to the turkey shed (Group E), another group (which included the affected ewe) were housed next to the duck and goose shed (Group H) with the remainder in the field adjacent to the garden (Group I). There were no linked premises nor commercial activities reported for this premises. Eggs and meat from the premises were only consumed by the keeper within the window of suspected infection.

Although the birds were housed indoors, overall biosecurity at the premises was assessed as poor. Structural deficiencies such as gaps in roofs, doors, and windows were identified, and no disinfectant footbaths were in use either at the entrance to the premises or between different sheds. The poultry sheds were not secure against wildlife ingress; entry points such as damaged windows, holes in the roof, and visible gaps in gates allowed potential access. Evidence of rat activity was also present, and the keeper reported observing a rat feeding on the head of a deceased chicken, although no samples were collected from rats on site. The birds were bedded daily, and the interiors of the poultry sheds were clean and dry. Mains-supplied drinkers were available within each shed. Although wild bird activity on site was reported to be low, sightings of wild birds flying overhead had been noted and there were bird feeders in the garden area near the sheds housing the poultry and sheep. Following the onset of avian mortality, recently deceased birds were stored in a garden waste bin.

The infection event in avian species began with initial signs of inappetence observed on 10^th^ February 2025 in both the ducks and chickens, accompanied by a decrease in egg production among the chickens (Figure 1c). By 16^th^ February 2025, one chicken and one duck had died, and one of the two geese was exhibiting neurological signs consistent with clinical disease, including head tremors. On 17^th^ February 2025, the clinically affected goose was found dead, and a further two chickens and one duck had succumbed to the infection. The owner reported suspicion of disease on 17^th^ February 2025 (Figure 1c). An official veterinarian from the Animal and Plant Health Agency (APHA) attended the premises the same day. Upon inspection, two of the four bird groups (Groups A and B) were found to be affected (Figure 1a). Two ducks displayed clinical signs suggestive of a notifiable avian disease (NAD); one exhibited “snicking” (a respiratory sign analogous to sneezing in humans), while the other appeared lethargic with ocular discharge. Additionally, one chicken was noted to be lethargic and was euthanised. A PM examination of that chicken revealed no significant gross lesions. However, NAD could not be ruled out, and samples were collected accordingly. These included oropharyngeal and cloacal swabs from 8 chickens (Group A), with 2 whole heads submitted for laboratory brain sampling; oropharyngeal and cloacal swabs from 11 ducks, along with 2 blood samples (from duck 18 and 19); and oropharyngeal and cloacal swabs from 1 goose (Group B). One of the clinically affected ducks died later on 17^th^ February 2025, and by the following day, the remaining clinically affected duck and the affected chicken had also died (Figure 1c).

Swab samples tested positive by RT–PCR for the influenza A virus (M-gene), H5 HPAIV (HP-H5), and N1 vRNA in 50% (4/8) of the chickens, 100% (11/11) of the ducks, and the single goose (1/1) (Supplementary Table 2). All swabs tested negative for type 1 avian paramyxovirus (APMV-1; referred to as Newcastle disease [ND] when virulent strains are present in poultry), as determined by RT–PCR targeting the L gene. Serological testing of duck blood samples by HAI revealed one (duck 18) out of two samples to be positive for H5 antibodies against clade 2.3.4.4b H5N1 antigen, with am HI titre of 1/64. These laboratory findings, in conjunction with the observed clinical signs, led to the official confirmation of H5N1 HPAI in avian species on the premises on 18th February 2025 (Figure 1c). Subsequent genetic sequencing identified all poultry genomes (Supplementary Table 3) as a clade 2.3.4.4b H5N1 HPAIV, belonging to the DI.2 genotype, in accordance with the genotype classification system established by the European Union Reference Laboratory (EURL) for Avian Influenza [49].

In accordance with protocols established in response to detections of HPAIV in captive dairy cattle in the USA, an epidemiological assessment was performed for this premises to determine whether any captive mammalian species present were at increased risk of exposure to HPAIV beyond the background level of risk posed by wild birds. Following this assessment, the sheep at this premises were triaged and sampled for diagnostic testing for the presence of HPAIV (Figure 1b).

On the 7^th^ of March, ten of the 26 sheep were sampled with nasal and rectal swabs being taken as well as a blood sample; the remaining animals were not sampled due to welfare concerns, as they were heavily pregnant. Nasal and rectal swabs collected from the sampled sheep (n = 10) (across Group H, G and F; Figure 1b) tested negative for vRNA by RT–PCR using the M-gene, H5-HP and N1 assays. Notably, serological testing of blood samples revealed that one ewe in Group H (Figure 1b) had detectable H5-specific antibodies, with a HI titre of 1:80. Two additional ELISAs also returned positive results from this serum, indicating the presence of antibodies reactive to NP and H5 antigens, respectively (see Supplementary Table 4). This initial positive serological detection led to repeat sampling of that sheep which was undertaken on the 14^th of^ March 2025 and although the animal was negative for vRNA from nasal and rectal swabs, the sheep was again positive for H5 reactive antibodies by HAIT (having a titre of 1/160), and the serum was positive by both ELISA tests. A composite milk sample (collected from both halves of the udder) tested positive for H5N1 HPAIV vRNA by M gene, HPH5 and N1 PCR assays. The milk sample was also assessed by competitive ELISA for NP and H5 and was positive in both tests.

Following the detection of H5-specific antibodies and H5N1 HPAIV vRNA in the milk, the affected ewe was culled on the 19^th^ of March 2025, and a full PM examination was undertaken. At this time, the ewe remained lactating, and milk was again expressed from both teats. No abnormalities in milk consistency or presence of clots were observed. Gross PM examination noted the left mammary gland to be slightly firm and mildly reddened; however, these findings were not considered clinically significant. Milk was collected post-euthanasia and post-swabbing, with samples from both halves of the udder pooled into a single container. Notably, this milk sample tested positive for both anti-NP and anti-H5 antibodies. All tissue samples taken at PME (n = 28) and swab samples (n = 12) (Supplementary Table 5) tested negative for HPAIV RNA by M gene, H5-HP, and N1 RT-PCRs. However, another milk sample from the ewe tested positive for H5N1 vRNA across all three RT–PCR assays, consistent with previous findings (Supplementary Table 5). Subsequently, tissue samples were further homogenised and reassessed for vRNA. Borderline detection of vRNA was observed in the homogenised right mammary gland tissue, but this result could not be consistently reproduced upon repeat RNA extraction and testing. Histological examination was conducted on these tissues. No lesions consistent with active mastitis or viral replication within the glandular tissue were observed, nor were there any definitive histological features indicative of prior viral exposure. Histopathological examination revealed areas of multifocal lymphoplasmacytic infiltration around alveoli and ducts in one half (left teat) (suggestive of chronic or residual inflammation). In contrast, multifocal regions of the other half exhibited acute neutrophilic alveolar exudation (more consistent with a recent inflammatory insult) (right teat), but not showing intraluminal epithelial sloughing and cellular debris in mammary alveoli described in active infection in cattle. IHC for the presence of viral antigen was also performed on mammary tissue sections; however, all sections were negative for viral antigen. Gram staining (Twort counterstain) of mammary gland samples did not reveal the presence of bacterial colonies (no further bacteriological studies were conducted).

Virus isolation was attempted on both H5N1 HPAIV positive milk samples using ECEs. However, infectious virus could not be isolated. We also attempted to sequester and concentrate potential virus in the milk samples using chicken RBCs. However, no infectious virus could be isolated following this treatment.

**Figure 2.**
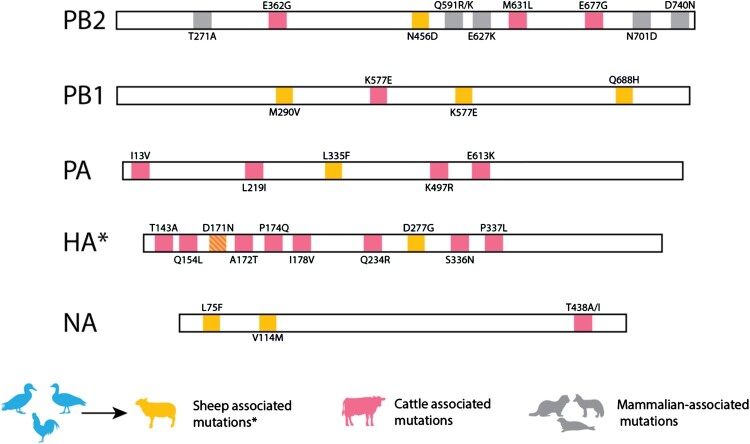
Genetic changes in sheep sequences. Amino acid mutations present in A/Sheep/England/023754/2025 consensus sequence when compared to WGS from associated poultry outbreak. Cattle mutations from [12,51,52]. NP, MP and NS segments not shown as there were no amino acid changes between avian and sheep sequence.

Full sequences were obtained from the milk sample for all segments except the PB1, which contained a large gap between nucleotides 333 and 786, as well as some smaller gaps between 190 and 330. The resulting sequence was compared to those derived from the infected birds and identified as a genotype DI.2 H5N1 clade 2.3.4.4b virus, clustering with other DI.2 isolates detected in GB during the same period (SI Figure 1). Further analysis of positive material from all infected birds (n = 21) (Supplementary Table 2) on the premises revealed >99.9% sequence identity across all segments. The viral sequence from the sheep contained nine amino acid substitutions relative to the avian viral sequences (Supplementary Table 6). Two changes were identified in the HA protein (D171N and D277G); neither is associated with increased zoonotic potential, although D171N has been previously reported in genotype B3.13 H5N1 sequences from cattle in the USA and may represent a ruminant-associated adaptation (Figure 2) [12,50]. Additional substitutions distinguishing the sheep and avian sequences included PB2 N456D, NA L75F and V114M, PA L335F and PB1 M290 V, K577E, and Q688H. Notably, previously reported cattle-adaptive mutations, such as PB2 E362G, M631L, and E677G; PB1 N642S; and PA A448S [12,51], were absent from the sheep-derived sequence. No mutations were detected in either the NP, MP or NS genes when compared with the avian reference sequence (Figure 2). The viral sequence from the sheep was also compared to publicly available sequences isolated from US goats in 2024. None of the sheep-associated mutations were present in the goat sequences.

## Discussion

The detection of clade 2.3.4.4b H5N1 vRNA in a sheep represents the first reported case of infection with this virus in this species globally, and the first detection in a ruminant outside of the USA. Previous detections of clade 2.3.4.4b H5N1 in mammalian species have generally been attributed to elevated environmental infection pressure, typically associated with large-scale mortality events in wild bird populations [2,3]. In such scenarios, transmission pathways are often apparent, particularly among scavenging species, where these mammals are presumed to have acquired infection through close contact with infected carcasses, most plausibly via the ingestion of dead or sick wild birds [2, 3].

However, this unusual detection raises the possibility of alternative routes of infection beyond the typical scavenging-behaviour related exposures. The ewe was co-located on an infected holding where chickens, ducks, and geese had tested positive for H5N1, and had been housed since December 2024 in a shed directly adjacent to the one housing both the ducks and geese (Group B). Initial avian testing revealed widespread vRNA shedding among the birds, via both cloacal and oropharyngeal routes. Importantly, the presence of infection in *Anseriformes* species (ducks and geese) increases the likelihood of a heavily contaminated environment, given their known capacity for extensive environmental viral shedding [30]. Multiple studies have shown that although chickens can efficiently transmit avian influenza viruses within dense commercial flocks, they typically shed lower quantities of infectious material when compared to ducks [30,52,53]. Experimental work consistently demonstrates that infected ducks release high viral loads into the environment, contaminating water and surfaces extensively. Based on this evidence, the lack of mitigating biosecurity measures in place between the poultry and the sheep managed by a single keeper who oversaw their daily management, and the common DI.2 sequence results isolated from the poultry and the ewe, it is reasonable to infer that the ewe may have acquired infection through indirect contact with the infected poultry shedding large amounts of HPAIV into the environment. However, another plausible hypothesis for source of HPAIV infection for the ewe considered was contact with infected wild birds or vermin. Indirect contact with wildlife was identified as the most likely source of infection for the poultry on this premises. This was supported by evidence including gaps in the shed doors and roofs which could have allowed for ingress by wild birds, associated faecal material or contaminated water, poor site biosecurity, evidence of rat activity on the premises, site features (including bird feeders and a pond) which may have attracted wild birds and the occurrence of both several other infected poultry premises and wild bird HPAIV detections in the surrounding area. It is possible that these same pathways and sources of infection are implicated for the ewe as well as the poultry. However, the weight of the above evidence reasonably supports the conclusion that poultry were more likely to be the source of infection for the ewe than wild birds.

Despite these findings, the precise route of infection in the ewe remains undetermined. However, several potential pathways are plausible, including (**i**) asymptomatic infection though the respiratory or oral routes via contaminated fomites, (**ii**) intramammary infection via contaminated material introduced into the teat by suckling lambs, or (**iii**) direct inoculation into the mammary gland as a result of the ewe lying in virus-contaminated material. Although none of these scenarios can be definitively ruled out, asymptomatic respiratory infection followed by systemic viral dissemination resulting in vRNA detection in milk appears unlikely. Recent studies have proposed that transfer of infectious virus between animals may occur during suckling with oral tissues of cattle supporting virus binding and replication [54], however, we did not detect infection of lambs during this study. Analysis of airborne virus present in commercial poultry sectors has demonstrated relatively low levels of virus present even within densely packed houses where assessed [55], but nevertheless various strains of H5N1 have been shown to transmit between avian species and/or between mammal species in experimental infections. While the infectious dose for sheep is unknown, considering the density of infected poultry present, the level of virus present in the air was likely low. In addition, if this had occurred, one would expect to observe evidence of systemic infection, such as seropositivity in other sheep or lambs; however, all animals tested were seronegative. HAIT was the frontline assay used to assess seroconversion and unfortunately, as samples were of a low volume, further assessment, such as virus neutralization tests, could not be undertaken. Of note, albeit anecdotal, the ewe with detectable vRNA in milk had a reported episode of mastitis on the 3^rd of^ March 2025. There was no evidence that prior unrelated infections may have reduced immune status in the affected ewe although with the report of mastitis in that animal in the period before this positive detection it is not possible to rule this out. No additional clinical signs or behavioural abnormalities were reported in the sheep following detection of infection in poultry. Interestingly, the detection of anti-NP and anti-H5 antibodies, indicating that the ewe was likely sampled during a phase of viral clearance, may explain the failure to isolate infectious virus from the collected samples.

For the second hypothesis (transmission via suckling lambs) to be plausible, one would expect some evidence of infection in the lambs, either through direct mucosal contact with infectious material or ingestion of contaminated milk. However, all lambs tested negative by both swab and serological assays, making this route of transmission less likely. Therefore, the third hypothesis appears most consistent with the available data. Experimental studies have shown that intramammary inoculation with both North American and European genotypes of H5N1 can lead to infection in cattle and is an efficient route of infection, albeit one that generally results in infection restricted to the udder [21]. In such cases, infection is frequently restricted to a single mammary quarter, without resulting in systemic dissemination. Unfortunately, in this case, milk samples were pooled from both teats, precluding any assessment of whether the infection was localized to one mammary gland. This limitation hinders the ability to definitively determine the anatomical extent of infection.

The histopathological findings were unable to provide clear evidence of active viral infection and where significantly different between both halves of the mammary gland, While in one of them subacute to chronic changes may have been consistent with a prior viral infection, the neutrophilic alveolar exudation in the other half would suggest a more recent insult or recent clearance, as no epithelial sloughing and cellular debris in the mammary alveoli associated with active infection [10] were seen. Although extensive sampling and examination of the mammary gland was conducted, some areas where virus was replicating may have been present elsewhere. Further data limitations arose due to logistical constraints associated with sampling pregnant animals. The detection of seropositivity in a single ewe highlighted the need for broader investigation within the flock. However, due to the flock's lambing status, comprehensive sampling of the entire flock could not be completed and only the initially seropositive ewe was subjected to further diagnostic evaluation at the time. All the sheep in the flock were ultimately sampled at least once, and all with negative results, although testing was protracted in the case of four ewes who did not lamb until late April and were only sampled on 26^th^ April 2025. Additionally, environmental sampling across the premises would have provided valuable insight into the degree of environmental contamination to which other mammals may have been exposed following the avian cull.

The viral sequence obtained from the ewe provides the only definitive evidence that replication occurred within this host. Comparative genomic analysis revealed several amino acid substitutions across multiple gene segments in the virus isolated from the sheep. These changes were absent in viral sequences derived from infected birds on the same premises, which exhibited a high degree of genetic identity among themselves. This divergence strongly suggests that the virus underwent replication and genetic adaptation within the ewe, consistent with host-specific evolutionary pressure. However, the functional significance of these mutations, particularly regarding viral fitness, host range, or transmissibility, remain unknown. Further virological and *in vivo* studies are required to assess the potential implications of these sequence changes for cross-species transmission and adaptation.

In conclusion, this study has demonstrated that it is possible for mammalian species co-located on an infected premises to become infected with these viruses through unusual infection routes and that appropriate risk assessments and both sampling and testing of mammals in such scenarios is important to understand where infection may have occurred. A recent report found that blood samples collected in 2024 from 220 sheep, which had grazed in areas affected by dead and diseased H5N1-infected pheasants in Norway during 2023, contained antibodies against H5 AIV [56]. Interestingly no clinical disease was reported in these sheep [56]. This supports the hypothesis that such spillover events may be more common than previously recognized. However, comprehensive surveillance for HPAIV is not routinely conducted in the ruminant sector. Should future cases arise whereby suspicion of infection of ruminant species is suspected then it would be critical to have better access to animals for sampling. On this occasion, the fact that sheep were in lamb significantly reduced our ability to sample the herd. Having access to a greater range of sample types, and volumes would allow confirmatory assessments to be made across the herd during the disease event. Certainly, with continued detections of high pathogenicity avian influenza across a broad range of wild and captive mammals globally it is important that a full characterization and assessment of spillover events is undertaken and shared with the global scientific community.

## Supplementary Material

Banyard_et_al_Sheep_MS_SUPPLEMENTARY_MATERIAL_post_review-clean.docx
