# Supplementary material for: Detection of clade 2.3.4.4b H5N1 high pathogenicity avian influenza virus in a sheep in Great Britain, 2025
Source: Emerg Microbes Infect. 2025 Sep 15;14(1):2547730. doi: 10.1080/22221751.2025.2547730 (PMC12439802; doi:10.1080/22221751.2025.2547730)
Supplement: Banyard_et_al_Sheep_MS_SUPPLEMENTARY_MATERIAL_post_review-clean.docx [file TEMI_A_2547730_SM9432.docx]

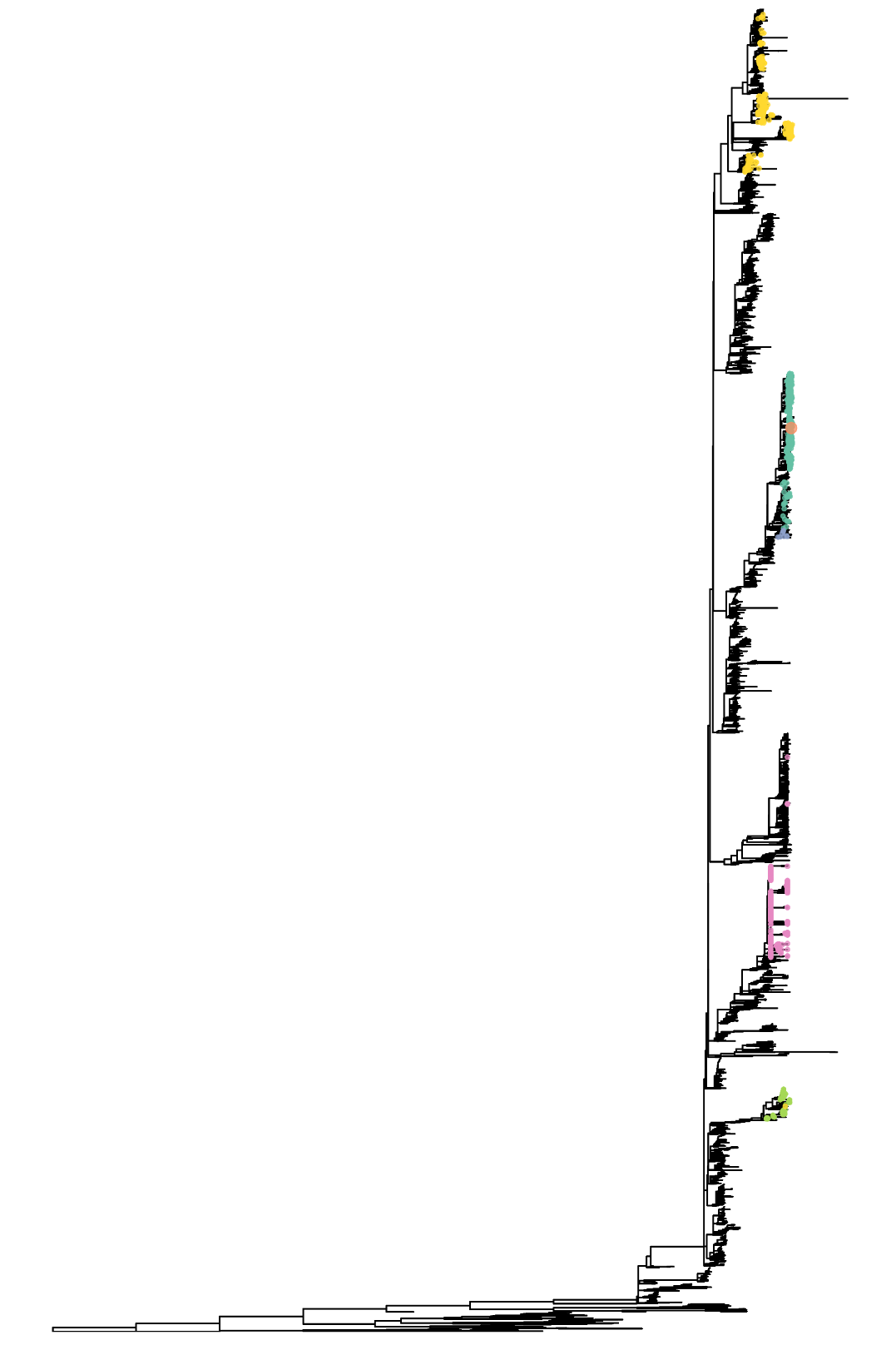

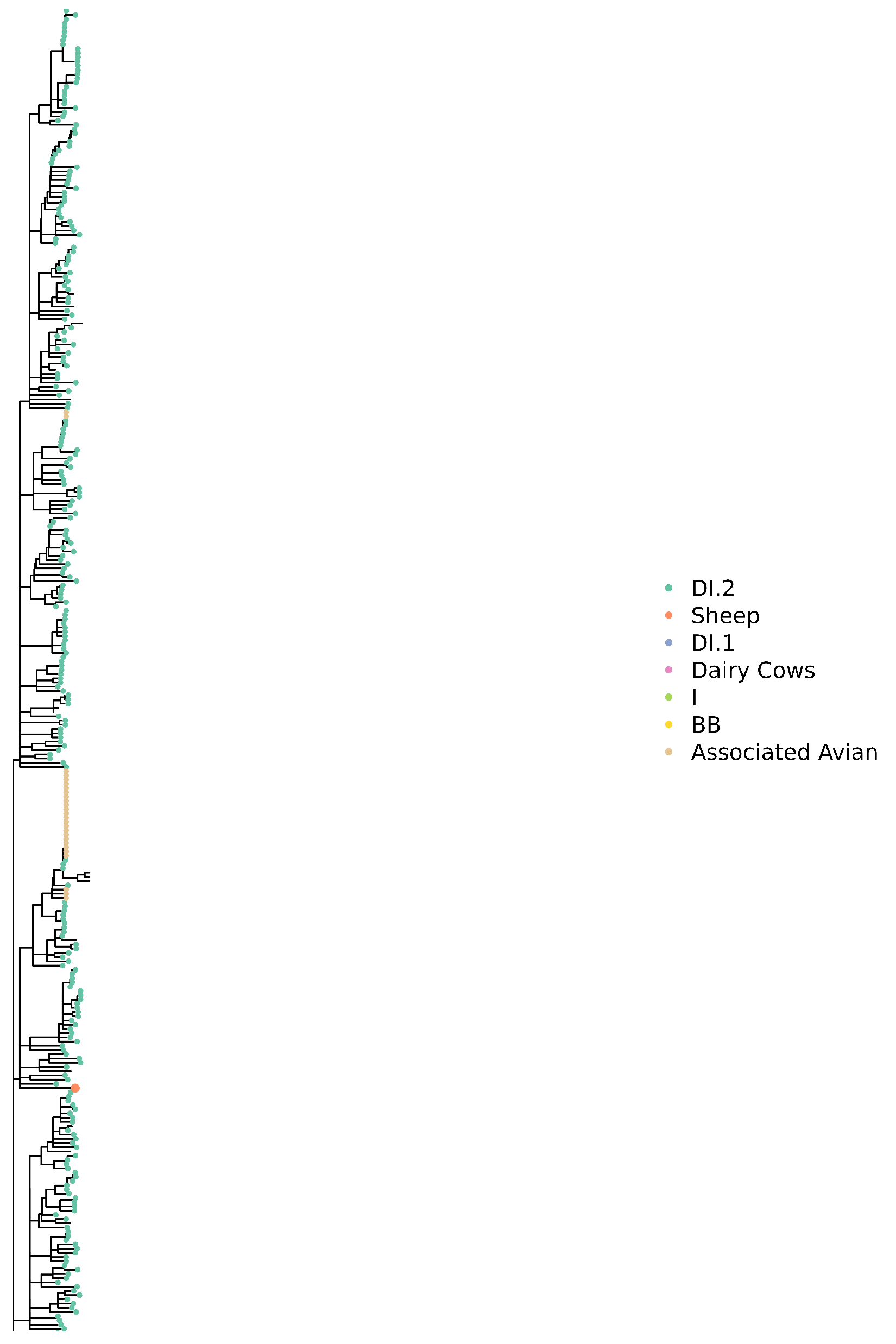

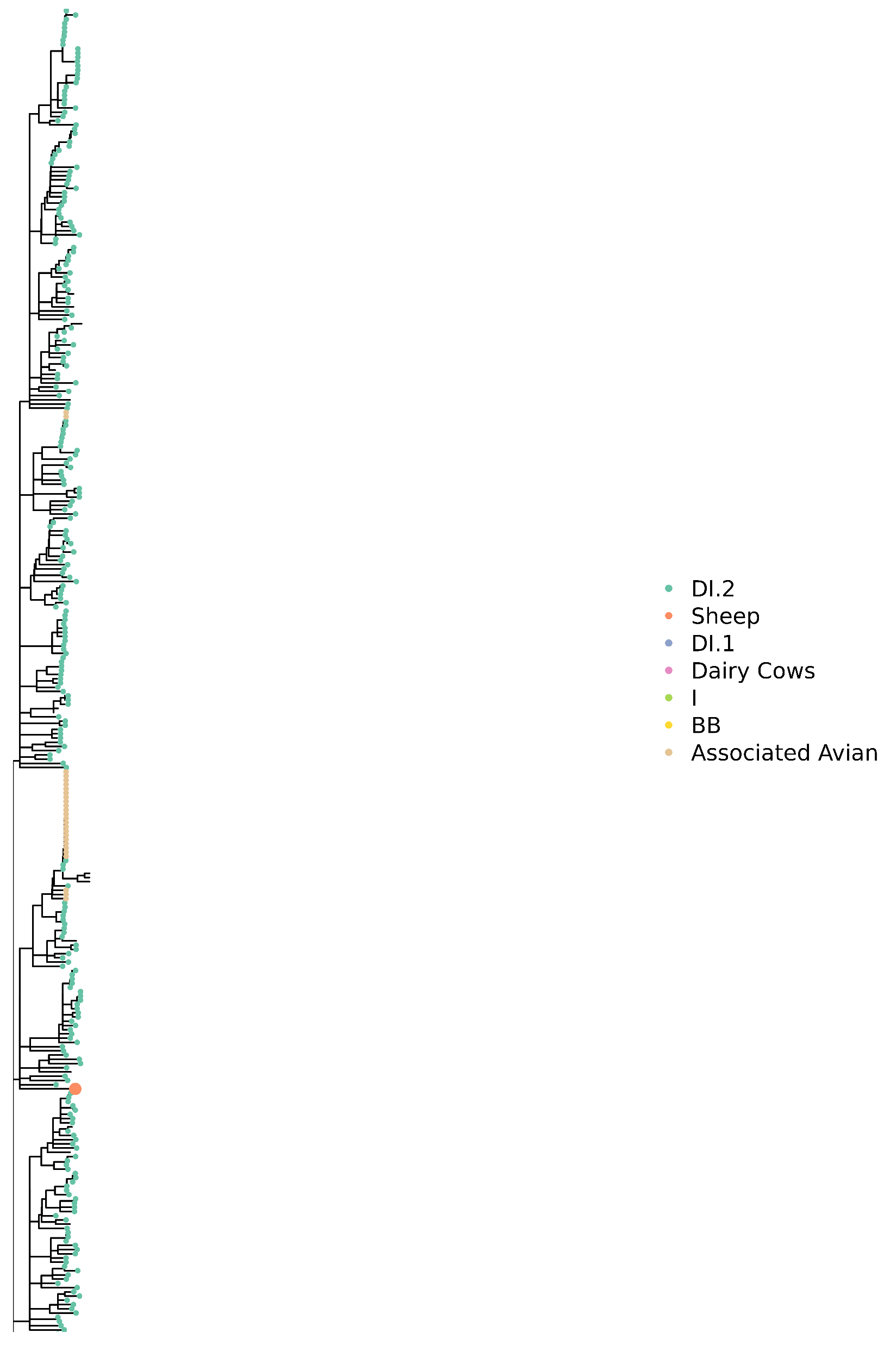


A/Sheep/England/023754/2025

**Supplementary Figure 1: Phylogenetic tree based on nucleotide sequence of HA gene.** Genotypes currently circulating in the UK highlighted along with sequences originating from cattle. Zoomed section highlighting sheep and associated poultry sequences from the same farm as the infected sheep. The viral sequence isolated from the sheep and all associated avian sequences are the DI.2 genotype.

**Supplementary Table 1:** Oligonucleotide primers used for cDNA synthesis and amplification for whole genome sequencing.

| **Name** | **Sequence (5’ to 3’)** | **Target Segment/gene** | **Reference** |
| --- | --- | --- | --- |
| Optil – F1 | TTACGCGCCAGCAAAAGCAG | All | [58, 59] |
| Optil – F2 | GTTACGCGCCAGCGAAAGCAGG | All |  |
| Optil – R1 | GTTACGCGCCAGTAGAAACAAG | All |  |
| PB1 - F1 | CWCARATACCWGCAGARATGCT | PB1 | APHA in house primers |
| PB1 – F403 | CAGACCTATGACTGGACATTGAA | PB1 |  |
| PA-F1 | AGC AAA AGC AGG TAC TGA TCC AA | PA |  |
| PA-R1493 | CTG CAT TTG CTT ATC ATT GGA ATC | PA |  |
| PA-F982 | GCC CAA CAT CGT GAA ACC ACA | PA |  |
| PA-R2233 | AGT AGA AAC AAG GTA CTT TTT TGG A | PA |  |
| S1 | AGCAGGGGTTCACTCTGTCA | HA1 | [57] |
| AS3 | GCTGGATGGCTCCTCGG | HA1 | APHA in house primers |
| J4 | GATTTCAACGACTATGAAGAACTG | HA1 |  |
| AS1 | AATGATGCGGCAGAGCAGAC | HA1 | [57] |
| KHA1 | CATCAATTTTGAGAGTAATG | HA1 | APHA in house primers |
| B2a | TTTTGTCAATGATTGAGTTGACCTTATTGG | HA1& 2 | [57] |
| AS4r | CCCCACAGTACCAAAAGATC | HA1 | APHA in house primers |
| AS2r | CCGAGGAGCCATCCAGC | HA1 |  |
| J3 | GATAAATTCTAGCATGCCATTCC | HA1 & 2 |  |
| H5-6 | GGGTACCACCATAGCAACGAGCAGGG | HA2 |  |
| AS6 | TACCCGCAGTATTCAGAAG | HA2 |  |
| J1c | AGTAGAAACAAGGGTGTT | HA2 |  |
| J2c | GGTGTTTTTAACTACAATCTGG | HA2 |  |
| TK-H5-2r | GGGAGCTCGCCACTGTTG | HA2 |  |
| KHA3 | TACCAACCGTCTACCATKCCYTG | HA2 |  |

**Supplementary Table 2:** Sequences generated in this study.

| **Species** | **Sample*** | **Collection Date** | **Subtype** | **Virus Strain Name** | **GISAID EPI ID** |
| --- | --- | --- | --- | --- | --- |
| Sheep | Milk | 2025-03-19 | H5N1 | A/sheep/England/023754/2025 | EPI_ISL_20089958 |
| Domestic Duck | Op swab | 2025-02-17 | H5N1 | A/domestic_duck/England/014229/2025 | EPI_ISL_20095048 |
| Domestic Duck | C swab | 2025-02-17 | H5N1 | A/domestic_duck/England/014230/2025 | EPI_ISL_20095072 |
| Domestic Duck | Op swab | 2025-02-17 | H5N1 | A/domestic_duck/England/014237/2025 | EPI_ISL_20095054 |
| Domestic Duck | Op swab | 2025-02-17 | H5N1 | A/domestic_duck/England/014241/2025 | EPI_ISL_20095055 |
| Domestic Duck | C swab | 2025-02-17 | H5N1 | A/domestic_duck/England/014242/2025 | EPI_ISL_20095056 |
| Domestic Duck | Op swab | 2025-02-17 | H5N1 | A/domestic_duck/England/014243/2025 | EPI_ISL_20095057 |
| Domestic Duck | Op swab | 2025-02-17 | H5N1 | A/domestic_duck/England/014245/2025 | EPI_ISL_20095058 |
| Domestic Duck | Op swab | 2025-02-17 | H5N1 | A/domestic_duck/England/014247/2025 | EPI_ISL_20095059 |
| Domestic Duck | C swab | 2025-02-17 | H5N1 | A/domestic_duck/England/014248/2025 | EPI_ISL_20095060 |
| Domestic Duck | C swab | 2025-02-17 | H5N1 | A/domestic_duck/England/014270/2025 | EPI_ISL_20095068 |
| Domestic Duck | Op swab | 2025-02-17 | H5N1 | A/domestic_duck/England/014232/2025 | EPI_ISL_20095052 |
| Domestic Duck | Op swab | 2025-02-17 | H5N1 | A/domestic_duck/England/014235/2025 | EPI_ISL_20095053 |
| Chicken | Op swab | 2025-02-17 | H5N1 | A/chicken/England/014249/2025 | EPI_ISL_20095062 |
| Chicken | C swab | 2025-02-17 | H5N1 | A/chicken/England/014250/2025 | EPI_ISL_20095063 |
| Chicken | Op swab | 2025-02-17 | H5N1 | A/chicken/England/014259/2025 | EPI_ISL_20095064 |
| Chicken | C swab | 2025-02-17 | H5N1 | A/chicken/England/014263/2025 | EPI_ISL_20095066 |
| Chicken | Op swab | 2025-02-17 | H5N1 | A/chicken/England/014265/2025 | EPI_ISL_20095067 |
| Goose | C swab | 2025-02-17 | H5N1 | A/goose/England/014272/2025 | EPI_ISL_20095070 |
| Goose | Op swab | 2025-02-17 | H5N1 | A/goose/England/014271/2025 | EPI_ISL_20095069 |
| Chicken | Brain | 2025-02-17 | H5N1 | A/chicken/England/014363/2025 | EPI_ISL_20095071 |
| Chicken | Op swab | 2025-02-17 | H5N1 | A/chicken/England/014262/2025 | EPI_ISL_20095065 |

* C = Cloacal; Op = Oropharyngeal

**Supplementary Table 3**: RT-PCR testing results for H5 HPAIV from captive chickens (Group A), ducks and geese (Group B) sampled on the 17 February 2025 where the sheep were co-located.

| **Group** | **Species** | **Bird ID** | **Sample Type** | **Interpretation from RT-PCR assay** | | | |
| --- | --- | --- | --- | --- | --- | --- | --- |
|  |  |  |  | **M-gene** | **H5-HP** | **N1** | **L-gene** |
| Group A | Chicken | 10 | Op | **Positive** | **Positive** | **Positive** | Negative |
|  |  |  | C | **Positive** | **Positive** | **Positive** | Negative |
|  | Chicken | 11 | Op | Negative | Negative | Negative | Negative |
|  |  |  | C | Negative | Negative | **Positive** | Negative |
|  | Chicken | 12 | Op | Negative | Negative | Negative | Negative |
|  |  |  | C | Negative | Negative | **Positive** | Negative |
|  | Chicken | 13 | Op | Negative | Negative | Negative | Negative |
|  |  |  | C | **Positive** | Negative | Negative | Negative |
|  | Chicken | 14 | Op | Negative | Negative | Negative | Negative |
|  |  |  | C | Negative | Negative | Negative | Negative |
|  | Chicken | 15 | Op | **Positive** | **Positive** | **Positive** | Negative |
|  |  |  | C | Negative | Negative | **Positive** | Negative |
|  | Chicken | 16 | Op | **Positive** | **Positive** | **Positive** | Negative |
|  |  |  | C | **Positive** | **Positive** | **Positive** | Negative |
|  | Chicken | 17 | Op | **Positive** | **Positive** | **Positive** | Negative |
|  |  |  | C | **Positive** | **Positive** | **Positive** | Negative |
|  | Chicken | 16,17 | Pooled Brain | **Positive** | **Positive** | **Positive** | Negative |
| Group B | Duck | 1 | Op | **Positive** | **Positive** | **Positive** | Negative |
|  |  |  | C | **Positive** | **Positive** | **Positive** | Negative |
|  | Duck | 2 | Op | **Positive** | **Positive** | **Positive** | Negative |
|  |  |  | C | Negative | Negative | **Positive** | Negative |
|  | Duck | 3 | Op | **Positive** | **Positive** | **Positive** | Negative |
|  |  |  | C | **Positive** | **Positive** | **Positive** | Negative |
|  | Duck | 4 | Op | **Positive** | **Positive** | **Positive** | Negative |
|  |  |  | C | **Positive** | **Positive** | **Positive** | Negative |
|  | Duck | 5 | Op | **Positive** | **Positive** | **Positive** | Negative |
|  |  |  | C | **Positive** | **Positive** | **Positive** | Negative |
|  | Duck | 6 | Op | **Positive** | **Positive** | **Positive** | Negative |
|  |  |  | C | **Positive** | **Positive** | **Positive** | Negative |
|  | Duck | 7 | Op | **Positive** | **Positive** | **Positive** | Negative |
|  |  |  | C | Negative | Negative | **Positive** | Negative |
|  | Duck | 8 | Op | **Positive** | **Positive** | **Positive** | Negative |
|  |  |  | C | Negative | Negative | **Positive** | Negative |
|  | Duck | 9 | Op | **Positive** | **Positive** | **Positive** | Negative |
|  |  |  | C | **Positive** | **Positive** | **Positive** | Negative |
|  | Duck | 18* | Op | **Positive** | **Positive** | **Positive** | Negative |
|  |  |  | C | **Positive** | **Positive** | **Positive** | Negative |
|  | Duck | 19* | Op | **Positive** | **Positive** | **Positive** | Negative |
|  |  |  | C | **Positive** | **Positive** | **Positive** | Negative |
|  | Goose | 20 | Op | **Positive** | **Positive** | **Positive** | Negative |
|  |  |  | C | **Positive** | **Positive** | **Positive** | Negative |

*blood collected for serology, Duck 18 gave positive serology result (1/64) in HI using H5N1 clade 2.3.4.4b antigen

OP- Oropharyngeal swabs; C- Cloacal swabs; L gene- Diagnostic test for avian paramyxovirus type -1

**Supplementary Table 4**: Serological assessment of sheep blood and milk samples taken from the infected premisses following detection of H5N1 HPAIV in poultry.

| **Collection date** | **Sheep identifier (sample type*)** | **HI** | **ELISA** | |
| --- | --- | --- | --- | --- |
|  |  | **H5N1-clade 2.3.4.4b^#^** | **ID Vet High path H5** | **ID Vet Multispecies** |
| 7^th^ March 2025 | Sheep 1 | <1/10 | Negative | Negative |
|  | Sheep 2 | <1/10 | Negative | Negative |
|  | Sheep 3 | <1/10 | Negative | Negative |
|  | Sheep 4 | <1/10 | Negative | Negative |
|  | Sheep 5 | <1/10 | Negative | Negative |
|  | Sheep 6 | <1/10 | Negative | Negative |
|  | Sheep 7 | **1/80** | **Positive** | **Positive** |
|  | Sheep 8 | <1/10 | Negative | Negative |
|  | Sheep 9 | <1/10 | Negative | Negative |
|  | Sheep 10 | <1/10 | Negative | Negative |
| 14^th^ March 2025 | Sheep 7 | **1/160** | **Positive** | **Positive** |
|  | Sheep 7 (milk) | ND | **Positive** | **Positive** |
| 19^th^ March 2025 | Sheep 7^$^ | **1/80** | **Positive** | **Positive** |
|  | Sheep 7 (milk) ^$^ | ND | **Positive** | **Positive** |
|  | Lamb 1^¥^ | <1/10 | Negative | Negative |
|  | Lamb 2^¥^ | <1/10 | Negative | Negative |
| 21^st^ March 2025^$^ | Sheep 1 | <1/10 | ND | ND |
|  | Sheep 2 | <1/10 | ND | ND |
|  | Sheep 3 | <1/10 | ND | ND |
|  | Sheep 4 | <1/10 | ND | ND |
|  | Sheep 5 | <1/10 | ND | ND |
|  | Sheep 6 | <1/10 | ND | ND |
|  | Sheep 7 | <1/10 | ND | ND |
|  | Sheep 8 | <1/10 | ND | ND |
|  | Sheep 9 | <1/10 | ND | ND |
|  | Sheep 10 | <1/10 | ND | ND |
|  | Sheep 11 | <1/10 | ND | ND |
|  | Sheep 12 | <1/10 | ND | ND |
|  | Lamb 1 | <1/10 | ND | ND |
|  | Lamb 2 | <1/10 | ND | ND |
|  | Lamb 3 | <1/10 | ND | ND |
|  | Lamb 4 | <1/10 | ND | ND |
|  | Lamb 5 | <1/10 | ND | ND |
|  | Lamb 6 | <1/10 | ND | ND |
|  | Lamb 7 | <1/10 | ND | ND |
| 1^st^ April 2025^$^ | Sheep 1 | <1/10 | ND | ND |
|  | Sheep 2 | <1/10 | ND | ND |
|  | Sheep 3 | <1/10 | ND | ND |
|  | Sheep 4 | <1/10 | ND | ND |
|  | Sheep 5 | <1/10 | ND | ND |
|  | Sheep 6 | <1/10 | ND | ND |
|  | Sheep 7 | <1/10 | ND | ND |
|  | Sheep 8 | <1/10 | ND | ND |
|  | Sheep 9 | <1/10 | ND | ND |
|  | Sheep 10 | <1/10 | ND | ND |
|  | Sheep 11 | <1/10 | ND | ND |
|  | Sheep 12 | <1/10 | ND | ND |
|  | Sheep 13 | <1/10 | ND | ND |
|  | Sheep 14 | <1/10 | ND | ND |
|  | Sheep 15 | <1/10 | ND | ND |
|  | Sheep 16 | <1/10 | ND | ND |
|  | Sheep 17 | <1/10 | ND | ND |
|  | Sheep 18 | <1/10 | ND | ND |
|  | Sheep 19 | <1/10 | ND | ND |
|  | Sheep 20 | <1/10 | ND | ND |
|  | Sheep 21 | <1/10 | ND | ND |
|  | Sheep 22 | <1/10 | ND | ND |
|  | Sheep 23 | <1/10 | ND | ND |
|  | Sheep 24 | <1/10 | ND | ND |
|  | Sheep 25 | <1/10 | ND | ND |

*Serum unless stated. ^¥^both lambs were offspring of sheep #7. The Sheep sera were diluted due to RDE treatment. ND = test not performed. ^#^ A/chicken/Wales/053969/2021 (H5N1) antigen. ^$^ Sheep 7 was culled on the 19^th^. Sampling numbers on the 21^st^ March and the 1^st^ April refer to new animals sampled and hence animal numbers are not aligned with the earlier samplings.

**Supplementary Table 5:** Summary of RT-PCR testing on sheep 7 samples taken from the infected premises following detection of H5N1 HPAIV in poultry.

| **Sampling Date** | **Sample type** | **RT-PCR assay** | | |
| --- | --- | --- | --- | --- |
|  |  | **M-gene** | **HP H5** | **N1** |
| 7^th^ March 2025 | Nasal swab | No Cq | No Cq | No Cq |
|  | Rectal swab | No Cq | No Cq | No Cq |
| 14^th^ March 2025 | Milk | **34.86** | **31.89** | **31.22** |
|  | Nasal swab | No Cq | No Cq | No Cq |
|  | Rectal swab | No Cq | No Cq | No Cq |
| 19^th^ March 2025  Swab and liquid samples PM | Milk | **34.02** | **33.55** | **32.98** |
|  | Blood (EDTA) | No Cq | No Cq | No Cq |
|  | Nasal swab | No Cq | No Cq | No Cq |
|  | Rectal swab | No Cq | No Cq | No Cq |
|  | External teat left swab | No Cq | No Cq | No Cq |
|  | External teat right swab | No Cq | No Cq | No Cq |
|  | Intra teat left swab | No Cq | No Cq | No Cq |
|  | Intra teat right swab | No Cq | No Cq | No Cq |
|  | Pharyngeal swab | No Cq | No Cq | No Cq |
|  | Skin & wool swab | No Cq | No Cq | No Cq |
|  | Tracheal swab 1 | No Cq | No Cq | No Cq |
|  | Tracheal swab 2 | No Cq | No Cq | No Cq |
| 19^th^ March 2025  Tissue samples PM | Abomasum | No Cq | No Cq | No Cq |
|  | Bladder | No Cq | No Cq | No Cq |
|  | Brain | No Cq | No Cq | No Cq |
|  | Bronchial section | No Cq | No Cq | No Cq |
|  | Colon | No Cq | No Cq | No Cq |
|  | Duodenum | No Cq | No Cq | No Cq |
|  | Gall Bladder | No Cq | No Cq | No Cq |
|  | Heart | No Cq | No Cq | No Cq |
|  | Ileum | No Cq | No Cq | No Cq |
|  | Jejunum | No Cq | No Cq | No Cq |
|  | Kidney | No Cq | No Cq | No Cq |
|  | Liver | No Cq | No Cq | No Cq |
|  | Lung | No Cq | No Cq | No Cq |
|  | Lymph nodes Pool | No Cq | No Cq | No Cq |
|  | Mammary gland -Left | No Cq | No Cq | No Cq |
|  | Mammary gland -Right | No Cq | No Cq | No Cq |
|  | Oesophagus | No Cq | No Cq | No Cq |
|  | Omasum | No Cq | No Cq | No Cq |
|  | Ovary | No Cq | No Cq | No Cq |
|  | Pancreas | No Cq | No Cq | No Cq |
|  | Pharyngeal Mucosa | No Cq | No Cq | No Cq |
|  | Reticulum | No Cq | No Cq | No Cq |
|  | Rumen | No Cq | No Cq | No Cq |
|  | Spleen | No Cq | No Cq | No Cq |
|  | Tracheal pool 1 | No Cq | No Cq | No Cq |
|  | Tracheal pool 2 | No Cq | No Cq | No Cq |
|  | Turbinate | No Cq | No Cq | No Cq |
|  | Uterus | No Cq | No Cq | No Cq |
| 19^th^ March 2025  Swab samples from lambs | Lamb 1- Oral Swab | No Cq | No Cq | No Cq |
|  | Lamb 1- Rectal Swab | No Cq | No Cq | No Cq |
|  | Lamb 2- Oral Swab | No Cq | No Cq | No Cq |
|  | Lamb 2- Rectal Swab | No Cq | No Cq | No Cq |
| 21^St^ March swab samples from sheep and lambs | Sheep 1-12 - Nasal swab | No Cq | No Cq | No Cq |
|  | Sheep 1-12 - Rectal swab | No Cq | No Cq | No Cq |
|  | Sheep 4,7,10 - Milk | No Cq | No Cq | No Cq |
|  | Lamb 1-7- Nasal swab | No Cq | No Cq | No Cq |
|  | Lamb 1-7- Rectal swab | No Cq | No Cq | No Cq |
| 1^st^ April 2025 Swab samples from remaining sheep and lambs | Sheep 1-25 Nasal | No Cq | No Cq | No Cq |
|  | Sheep 1-25 Rectal | No Cq | No Cq | No Cq |
|  | Sheep 1,2,4 Milk Left teat | No Cq | No Cq | No Cq |
|  | Sheep 1,2,3,4 Milk Right teat | No Cq | No Cq | No Cq |

**Supplementary Table 6:** Amino acid differences detected in the viral RNA detected in the sheep compared to the sequence derived from the birds on the infected premises.

| **Segment** | **Amino acid position** | **Avian sequences*** | **Sheep #7 (milk)** |
| --- | --- | --- | --- |
| PB2 | 456 | N | D |
| PA | 335 | L | F |
| NA | 75 | L | F |
|  | 114 | V | M |
| HA | 171^a^ (155^b^ / 159^c^) | D | N |
|  | 277^a^ (261^a^ / 264^b^) | D | G |
| PB1 | 290 | M | V |
|  | 577 | K | E |
|  | 688 | Q | H |

*Compared to all avian sequences derived from the infected premises; ^a^ immature H5 HA numbering; ^b^ mature H5 HA numbering; ^c^ mature H3 numbering.

**Supplementary Table 7:** Sequence coverage and mean depth for all viral sequences generated in this study

| **GISAID EPI ID** | **Virus Strain Name** | **Coverage*** | **% bases with ≥10x coverage*** |
| --- | --- | --- | --- |
| EPI_ISL_20089958 | A/sheep/England/023754/2025 | 1556 | 97.5 |
| EPI_ISL_20095048 | A/domestic_duck/England/014229/2025 | 6919 | 100 |
| EPI_ISL_20095072 | A/domestic_duck/England/014230/2025 | 3846 | 100 |
|  | A/domestic_duck/England/014237/2025 | 380 | 100 |
| EPI_ISL_20095055 | A/domestic_duck/England/014241/2025 | 2709 | 100 |
|  | A/domestic_duck/England/014242/2025 | 4085 | 100 |
| EPI_ISL_20095057 | A/domestic_duck/England/014243/2025 | 2461 | 100 |
| EPI_ISL_20095058 | A/domestic_duck/England/014245/2025 | 1753 | 100 |
| EPI_ISL_20095059 | A/domestic_duck/England/014247/2025 | 3791 | 100 |
| EPI_ISL_20095060 | A/domestic_duck/England/014248/2025 | 1674 | 100 |
| EPI_ISL_20095068 | A/domestic_duck/England/014270/2025 | 2498 | 100 |
| EPI_ISL_20095052 | A/domestic_duck/England/014232/2025 | 380 | 100 |
| EPI_ISL_20095053 | A/domestic_duck/England/014235/2025 | 5598 | 100 |
| EPI_ISL_20095062 | A/chicken/England/014249/2025 | 7816 | 100 |
| EPI_ISL_20095063 | A/chicken/England/014250/2025 | 7471 | 100 |
| EPI_ISL_20095064 | A/chicken/England/014259/2025 | 1982 | 100 |
| EPI_ISL_20095066 | A/chicken/England/014263/2025 | 3799 | 100 |
| EPI_ISL_20095067 | A/chicken/England/014265/2025 | 5507 | 100 |
| EPI_ISL_20095070 | A/goose/England/014272/2025 | 6694 | 100 |
| EPI_ISL_20095069 | A/goose/England/014271/2025 | 6165 | 100 |
| EPI_ISL_20095071 | A/chicken/England/014363/2025 | 6472 | 100 |
| EPI_ISL_20095065 | A/chicken/England/014262/2025 | 6044 | 100 |

*****Averaged across all 8 segments.
